# Aberrant Functional Connectivity in the Default Mode and Central Executive Networks in Subjects with Schizophrenia – A Whole-Brain Resting-State ICA Study

**DOI:** 10.3389/fpsyt.2015.00026

**Published:** 2015-02-26

**Authors:** Harri Littow, Ville Huossa, Sami Karjalainen, Erika Jääskeläinen, Marianne Haapea, Jouko Miettunen, Osmo Tervonen, Matti Isohanni, Juha Nikkinen, Juha Veijola, Graham Murray, Vesa J. Kiviniemi

**Affiliations:** ^1^Department of Radiology, Medical Research Center, Oulu University Hospital, Oulu, Finland; ^2^Department of Psychiatry, Medical Research Center, Oulu University Hospital, Oulu, Finland; ^3^Department of Oncology, Medical Research Center, Oulu University Hospital, Oulu, Finland; ^4^Department of Psychiatry, University of Cambridge, Cambridge, UK

**Keywords:** fMRI, ICA, resting state, schizophrenia, default mode network, central executive network, caudate nucleus

## Abstract

Neurophysiological changes of schizophrenia are currently linked to disturbances in connectivity between functional brain networks. Functional magnetic resonance imaging studies on schizophrenia have focused on a few selected networks. Also previously, it has not been possible to discern whether the functional alterations in schizophrenia originate from spatial shifting or amplitude alterations of functional connectivity. In this study, we aim to discern the differences in schizophrenia patients with respect to spatial shifting vs. signal amplitude changes in functional connectivity in the whole-brain connectome. We used high model order-independent component analysis to study some 40 resting-state networks (RSN) covering the whole cortex. Group differences were analyzed with dual regression coupled with y-concat correction for multiple comparisons. We investigated the RSNs with and without variance normalization in order to discern spatial shifting from signal amplitude changes in 43 schizophrenia patients and matched controls from the Northern Finland 1966 Birth Cohort. Voxel-level correction for multiple comparisons revealed 18 RSNs with altered functional connectivity, 6 of which had both spatial and signal amplitude changes. After adding the multiple comparison, y-concat correction to the analysis for including the 40 RSNs as well, we found that four RSNs showed still changes. These robust changes actually seem encompass parcellations of the default mode network and central executive networks. These networks both have spatially shifted connectivity and abnormal signal amplitudes. Interestingly the networks seem to mix their functional representations in areas like left caudate nucleus and dorsolateral prefrontal cortex. These changes overlapped with areas that have been related to dopaminergic alterations in patients with schizophrenia compared to controls.

## Introduction

Schizophrenia is a psychotic disorder that manifests itself by altering patients’ mental function, emotional life, and functional capacity. Common symptoms consist of auditory hallucinations, paranoid delusions, and disorganized speech and thinking. Neurobiological changes of the disease itself are currently believed to be related to disturbances in functional connectivity between brain regions (1–5).

A great number of studies have been conducted using blood oxygen level-dependent (BOLD) functional magnetic resonance imaging (fMRI) signal, mostly assessing task-related activation of the brain. However, there has been an increasing interest in studying alterations in resting-state fMRI (rsfMRI). The advantage of rsfMRI is that patients do not have to perform any particular task other than maintain consciousness and lay still, as patients may be unable to perform more complex task or lay still while doing them. The rsfMRI studies show robust anomalies in the default mode network (DMN) and other intrinsic, i.e., resting-state networks (RSN) in schizophrenia patients (6–13).

Literature on schizophrenia research on resting-state data suggests that especially DMN, a task-positive frontoparietal network (TPN), and the salience network (SN) are involved. The DMN is considered to be involved in self-related/internally oriented processes (14) and it consists of the anterior and posterior cingulate cortex (PCC), the medial prefrontal cortex (mPFC), the precuneus, the parahippocampal areas, and the inferior parietal cortices. It is particularly active during “rest” and is deactivated during the performance of a variety of cognitive tasks when TPN becomes activated by the task (15–17). Williamson and Allman (12) listed 25 studies that show reduced task-related suppression of the DMN and also noted that both increased (18, 19) and decreased (20–23) connectivity have been found in the DMN. This could be explained with the assumption that the DMN is not one network, but a compilation of networks, with only a few of them being affected by the disease (12, 24). Karbasforoushan and Woodward (9) reported that in their review of resting-state anomalies in schizophrenia, the majority of investigations reported hyper-connectivity of the DMN.

The task-positive network is an anti-correlating network compared to DMN responsible for high-level cognitive functions, notably the control of attention, working memory, and executive task-performance (25, 26). More recent studies by Sridharan et al. (27), and Menon and Uddin (28) have brought up the term central executive network (CEN) anchored in the dorsolateral prefrontal cortex (dlPFC) and posterior parietal cortices (PPC), which is similar to TPN (26–28). Depending on the researcher, CEN is often referred to as the executive-control network (ECN), or the frontoparietal network (FPN), and is sometimes divided to CEN and the dorsal attention network (DAN) [i.e., Ref. (29)]. The DAN consists of the intraparietal sulcus/superior parietal lobule, frontal eye fields, and extrastriate visual areas. Further discussion of CEN in this paper includes DAN in line with the work of Sridharan et al. (27) and Menon and Uddin (28).

The CEN is involved in goal-directed/externally oriented tasks. Altered connectivity within a FPN has been reported in several studies (23, 29–34). Lui et al. (35) did not find abnormal dlPFC connectivity in antipsychotic naïve first-episode patients. Altogether, the severity of illness is indicated to be correlated with differences in functional connectivity (15, 23, 33, 36–42).

Some researchers usually regard DMN and CEN as the only areas of RSNs, but actually the whole-brain cortex can be parcelled into at least 42 functional networks using independent component analysis (ICA) (24, 43). During past few years, ICA has become one of the most often used blind source separation tools for resting-state brain networks since it effectively separates noise from neurophysiological noise sources (44). Changes in functional connectivity in other anatomical areas of the other RSNs have also been reported in schizophrenia. Given the large number of RSNs, and the wealth of literature on separate regional alterations in schizophrenia resting-state BOLD studies, it seems logical that several functionally connected brain networks can be affected by schizophrenia simultaneously. Previous resting-state reports have often focused either on no specific functional network with global brain signal analysis tools, or, focused on one single network, due to statistical limitations in comparing multiple networks simultaneously. Graph theoretical studies do analyze multiple drawn brain atlas regions simultaneously, but tend to analyze data on a graph theoretical frame without functional parcellation of areas (45, 46). Hand drawn regions of interest or atlas-based regions of interest are not accurate parcellations of brain functional connectome (47). These methods often either focus on signal properties or anatomical differences, but rarely on both.

Our group has developed techniques that can detect fine grained functional parcellations of the brain networks with high model order ICA; for example, DMN can be parcelled into three networks (48). The high model order ICA combined with a more rigorous correction for multiple comparisons (y-concat) enables the analysis of all detected functionally independent brain networks simultaneously (49). Moreover, by using different *variance normalization* options in dual regression, one can assess whether the difference is a more spatially shifted activity or whether the difference originates from amplitude changes in signals reflecting functional connectivity (49, 50).

In this study, we explored the whole-brain functional connectome with the high model order ICA method in subjects with schizophrenia and matched birth cohort controls. We compared the high model order functional connectome parcellations of schizophrenia subjects with matched population controls using a dual regression approach. We aimed to assess (a) if multiple differences between the groups can be detected simultaneously in several RSNs, and (b) whether the group differences in functional connectivity are due to anatomical shift or signal amplitude change in functional activity mediating connectivity. We assessed the statistical significance of the group difference results by y-concatenated threshold-free cluster enhanced (TFCE) randomize analysis (43, 51, 52). Finally, we compared our whole-brain analysis results to the existing literature on functional connectivity alterations that had been detected previously in schizophrenia.

## Materials and Methods

### The Northern Finland 1966 birth cohort

All subjects of this study are members of the Northern Finland 1966 Birth Cohort (NFBC1966, http://kelo.oulu.fi/NFBC/index.html). The NFBC1966 is an unselected population birth cohort ascertained during mid-pregnancy. The cohort is based upon 12,058 children with an expected date of birth during 1966 (53). Permission to collect data was obtained from the Ministry of Social and Health Affairs. The Ethical committee of the Northern Ostrobothnia Hospital District (Oulu, Finland) has accepted and continuously supervised the study design.

### Study sample

NFBC1966 members with a possible psychosis were identified using the following sources:

1. the Finnish Hospital Discharge Register (FHDR) 1982-2008 (FHDR covers all hospitals, both mental and general, and beds in local health care centers throughout the country);
2. the Finnish Social Insurance Institutions (SII) register data until the end of 2008 (i.e., sick leave or disability pension due to psychosis, or the right for reimbursement for psychoactive medication);
3. the cohort questionnaire at 31 years of age [self reporting either a physician detected psychosis, or the regular and substantial use of antipsychotics (more than 300 mg chlorpromazine equivalents daily)].

Altogether 266 members with a possible psychosis were invited to a field study (2008–2010). A random sample of 450 presumably non-psychotic controls was selected from the NFBC1966 and invited to the field study. Participation figures of the field study were 107 (40%) in the psychosis group and 194 (43%) in the control group. Fifty-five subjects in the psychosis group were classified as having schizophrenia.

Exclusion criteria included organic psychosis (*n* = 7), a history of head trauma with a loss of consciousness for over 30 min (*n* = 4), major neurologic disorder (e.g., multiple sclerosis or epilepsy with antiepileptic medication, *n* = 2). Failure in completing the MRI scan resulted in the exclusion of two subjects with schizophrenia from the present study. Simultaneous non-psychotic mental disorders were not considered as exclusion criteria in either of the study groups.

Participants answered questionnaires and underwent psychiatric interviews, cognitive testing, and an MRI of the brain (54, 55). All participants gave written informed consent and were interviewed using a Structured Clinical Interview for DSM-IV (SCID-I) (56). In addition to the 55 subjects diagnosed as having schizophrenia based on the three above mentioned sources, three cases were included in the schizophrenia group based on the clinical interviews conducted during the field study. These individuals were originally invited based on some other psychotic disorder.

In the end, a total of 43 subjects with schizophrenia and a successful MRI scan formed the case group of the present study. In the present study, an equal amount (*n* = 43) of non-psychotic subjects was randomly chosen for the control group. The controls were matched by gender and by handedness. Because both groups were selected from the same birth cohort, the matching by age occurred as a by-product. Most (36/43) of the schizophrenia patients have current antipsychotic medication, 8 patients having benzodiazepines and 11 antidepressants (see Table 1).

**Table 1:** **Demographics of the participants of the study**.

	M	SD	M	SD
Gender males/females	26/17		26/17	
Age	43.1	0.7	43.5	0.8
PANNS score total	71	26	n/a	n/a
Positive	16	7.5	n/a	n/a
Negative	19	10	n/a	n/a
Medication (CPZ equivalent dose mg/day)	315	286	n/a	n/a
Duration of illness	18.8	11.2	n/a	n/a

*CPZ, chlorpromazine equivalents; PANSS, Positive and Negative Syndrome Scale*.

### Data acquisition and pre-processing

Resting-state BOLD data were collected on a GE Signa 1.5 T whole body system with an eight channel receive coil, using an EPI GRE sequence (TR 1800 ms, TE 40 ms, 280 time points, 28 oblique axial slices, slice thickness 4 mm, inter-slice space 0.4, covering the whole brain, FOV 25.6 cm × 25.6 cm, with 64 × 64 matrix, parallel imaging factor 2, and a flip angle of 90°). T1-weighted scans were imaged using a 3D FSPGR BRAVO sequence (TR 12.1 ms, TE 5.2 ms, slice thickness 1.0 mm, FOV 24.0 cm, matrix 256 × 256, and flip angle 20°, and NEX 1) in order to obtain anatomical images for co-registration of the fMRI data to standard space coordinates.

Head motion in the fMRI data was corrected using multi-resolution rigid body co-registration of volumes, as implemented in FSL 3.3 MCFLIRT software (57). Brain extraction was carried out for motion corrected BOLD volumes with optimization of the deforming smooth surface model, as implemented in FSL 3.3 BET software (58). Then, the BOLD volumes were spatially smoothed with a Gaussian kernel (7.5 mm FWHM) and voxel time series were high-pass filtered using a Gaussian linear filter with a 100 s cutoff. The FSL 4.1.4 fslmaths tool was used for these steps. Multi-resolution affine co-registration, as implemented in the FSL 4.1.4 FLIRT software (57), was used to co-register mean non-smoothed fMRI volumes to 3D FSPGR volumes of corresponding subjects, and 3D FSPGR volumes to the Montreal Neurological Institute (MNI) standard structural space template (MNI152_T1_2mm_brain template included in FSL). However, for computational reasons pertaining to later analysis steps, 4 mm resolution was retained after spatial normalization.

#### ICA analysis

We have previously addressed the influence of ICA model order selection on the patterns of between-group differences (48). Based on this, we assessed functional connectivity at the local optimum hierarchical level of model order 70. ICA analysis was carried out using FSL 4.1.4 MELODIC software implementing probabilistic-independent component analysis (PICA) (59). The multisession temporal concatenation tool in MELODIC was used to perform PICA-related pre-processing and data conditioning in the group analysis setting. ICA using high model order of 70 independent component maps (IC maps) was applied to detect RSNs. The IC maps were thresholded using an alternative hypothesis test based on fitting a Gaussian/gamma mixture model to the distribution of voxel intensities within spatial maps (60) and controlling the local false-discovery rate at *p* < 0.5. The data for between-subject analysis of the resting data were obtained using a regression technique (dual regression) that allows voxel-wise comparisons of data (48, 52, 61–64). In dual regression, the spatial maps of group ICA are regressed to the pre-processed BOLD signal data of each individual to find spatial representations of ICs at an individual level. Then, a derived temporal signal, measuring the fit of the given spatial map to the BOLD signal is again regressed to the data, whence the name dual regression. This gives the possibility to assess group-derived RSNs at the individual level. The RSNs were identified by two neuroradiologists (Harri Littow and Vesa J. Kiviniemi) using previous selection criteria (24). Dual regression was performed using all the ICs in order to effectively separate motion, pulsation, and other physiological sources. However, in the second level correction for multiple comparisons due to several RSNs, only RSN-related ICs were used. The dual regression was performed using both non-normalized (des_norm = 0) and normalized signal variance (des_norm = 1 in FSL). This is important to notice since non-normalized results reflect spatial connectivity changes and normalized variance results reflect signal power changes in the RSNs, respectively.

#### Statistical analysis and correction for multiple comparisons at the whole-brain level

In order to measure functional connectivity of several RSNs covering the whole brain, one needs to correct for multiple comparisons both at the voxel level and over several RSNs. Especially at high model orders, the commonly used voxel-level correction does not adjust for the risk of a type 1 error (false positives) induced by increasing the number of components tested simultaneously. We have previously developed a method that enabled the correction for multiple comparisons due to simultaneous assessment of several RSNs (49). We call this method *y-concatenation (y-concat) correction* due to its nature of concatenating data in a *y*-direction and the term is used from here on.

Initially, between-group statistical difference was assessed non-parametrically using permutation testing implemented in FSLs Randomize tool (v2.1), incorporating also TFCE (43) for cluster-like statistic and use of maximal statistics for multiple comparisons correction. This involved deriving null distributions of TFCE-values for the contrasts, reflecting the between-group effects by performing 10,000 random permutations of group labels, and testing the difference between groups against the distribution of maximal statistic values from all permutations (65).

**Figure 1:**
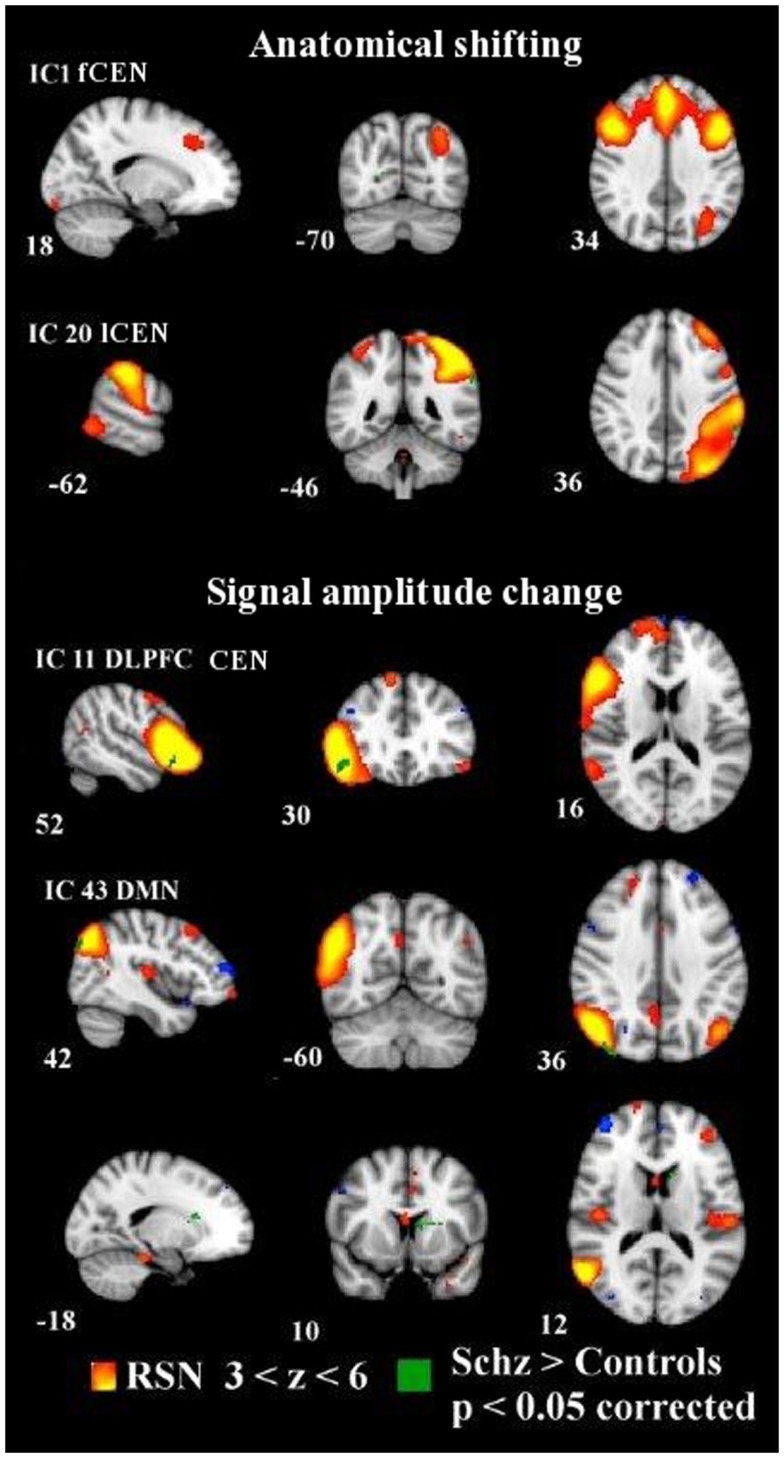
**Dual regression analysis with non-normalized variance in IC1 and IC20 showing anatomically shifted differences between groups**. Normalized variance analysis below shows in IC11 and IC43 signal amplitude changes between groups. Changes overlaid on MNI152 template with coordinate planes in white. Color encoding of individual RSN in hot colors with *z*-score thresholds and in green the y-concatenated differences between groups. fCEN, frontal central executive network; lCEN, left central executive network; rDLPFC, right dorsolateral prefrontal cortex; DMN, default mode network.

In the second level, y-concat correction was performed by taking the temporally concatenated subject-specific RSN maps derived from the initial dual regression, and concatenating the RSN maps in the *y*-direction (Figure 1). Then, statistical analysis using permutation testing (implemented in the FSLs randomize tool, 10,000 random permutations) was performed on the resulting concatenated map (86 subjects temporally concatenated and 39 RSN maps spatially concatenated). After brain extraction, voxels outside the brain are set to zero and consequently non-zero voxels in maps remain spatially disjointed after spatial concatenation. Therefore, the computation of the TFCE-statistic (and other cluster-related statistics) in individual maps remain unaffected with respect to concatenation. In practice, the second level (inter-RSN) multiple comparison correction computes a maximal statistic for each permutation over TFCE-statistics of all voxels of the concatenated maps (relative to the initial analysis that computes TFCE-statistics in each map separately). Then, the resulting distribution of maximal values is used to derive threshold levels for *p*-values.

The mean relative motion was regressed from the data in order to further minimize motion. In addition to motion, CSF/blood flow, pulsation, respiration, etc., nuisance ICs were discarded prior to y-concat correction in order to specifically sensitize the analysis to RSNs. The resulting statistical between-group difference maps were then divided back into 39 ICs from the concatenated data, thresholded at p < 0.05 (corrected now for family-wise errors within and between all concatenated RSN maps), and, resampled into 2 mm. The Juelich histological atlas (66) and the Harvard–Oxford cortical and subcortical atlases (Harvard Center for Morphometric Analysis) provided with the FSL software were used to identify the anatomical characteristics of both RSNs and between-group differences.

## Results

In this study, before the y-concatenation, our findings between schizophrenia patients and controls at the voxel level were remarkably extensive. We found 18 RSNs with significant (p < 0.05, corrected for family-wise errors for each RSN map separately at voxel level) changes between groups. Thirteen RSNs showing increased functional signal amplitude connectivity and 10 RSNs with anatomically aberrant activity in patients with schizophrenia compared to controls. ICs 20, 43, 49, 56, and 60 overlapped and had both anatomic and amplitude differences between groups (see Figures S1 and S2 and Table S1 in Supplementary Material).

We found anatomically aberrant resting-state connectivity in the visual cortex BA18 (IC6) and BA17 L (IC60), juxtapositional lobule cortex (IC13), supramarginal gyrus and infraparietal lobule (IC20), paracingulate gyrus (IC22), postcentral gyrus (IC42), lateral occipital cortex (IC43, IC56), left caudatus (IC43), middle temporal gyrus (IC49), and anterior cingulate gyrus (IC63).

Higher signal amplitude in schizophrenia patients was found in the middle frontal gyrus within the frontal parts of the CEN (IC1), inferior frontal gyrus (IC11, IC46), frontal orbital cortex (IC11), bilateral primary auditory cortex, superior temporal gyrus and thalamus (IC19), inferior parietal lobule (IC20, IC43), inferior supramarginal gyrus (IC20), precentral gyrus (IC29), frontal pole (IC31), lateral occipital cortex (IC43, IC56, IC60), left insula (IC43), left caudatus (IC43), juxtapositional lobule cortex (IC43), Broca’s area BA45 (IC46), cerebellum (IC49), and the premotor cortex (IC67).

Correction for multiple RSN comparisons with y-concat diminished the number of altered networks markedly. After the y-concat procedure, higher signal amplitude was detected within the schizophrenia group in IC11 and IC43. IC11 consists mainly of a right dlPFC with functional connectivity to the left superior frontal gyrus, left angular gyrus, and left dlPFC. Schizophrenia patients have greater signal amplitude in the core area of right dlPFC within the network depicted by IC 11. IC43 is the DMN variant focused on the angular gyrus and consists of bilateral inferior parietal lobules and portions of bilateral cingulate gyri. The right side shows an extension to the temporal lobe. The schizophrenia group has greater amplitude in the right inferior parietal lobule, in bilateral medial frontal gyri (BA6) and in the left caudate nucleus compared to the control group (see Figure 1; Table 2).

**Table 2:** **Group comparison of independent components after second level (inter-RSN) *Y*-concatenation correction**.

	IC #	Vox	Anatomical location of maximal change	Mean *T*-score	Std	Min	Max	Max coordinates		
								*X*	*Y*	*Z*
**Spatial**
*fCEN*	1	1	Right medial visual cortex	5.68	–	5.68	5.68	36	28	42
*lCEN*	20	11	Left inferior parietal lobule	3.67	0.26	3.34	4.28	76	40	52
**Amplitude**
*rDLPFC (CEN)*	11	79	Right dorsolateral prefrontal cortex	3.54	0.29	3.18	4.48	18	76	26
*DMN*	43	96	Left caudate	3.95	0.33	3.43	4.86	28	20	52

*Co-ordinates indicating the centroid of the cluster of altered brain activity*.

Anatomically, we found two ICs with spatial differences between the subjects with schizophrenia and the controls (a.k.a dual regression without variance normalization). The subjects with schizophrenia show slightly wider connectivity in the left inferior parietal lobule within the left CEN (lCEN, IC20) consisting of the left PPC and left superior frontal gyrus. In addition, the schizophrenia group has a very small spot of aberrant connectivity in the right medial visual cortex in connection with the frontal parts of the CEN (IC1), which consists of anterior bilateral paracingulate gyri, bilateral medial frontal gyri, and left inferior parietal lobule. Notably, the control group neither exhibited a greater signal amplitude nor a greater spatial extent of connectivity in any of the ICs (see Figure 1).

## Discussion

In the present paper, we demonstrate that dual regressed ICA can be used to study all the networks of the human brain connectome in rsfMRI simultaneously. Our results show that variance normalization can indeed help in discerning some of the signal amplitude changes in functional connectivity from spatial shifts of activity within the networks. Patients with schizophrenia have increased signal amplitude changes in the functional connectivity of the frontal CEN with the dlPFC compared to controls. Also the right parietal lobe (BA 19), and the bilateral superior medial frontal cortices (BA6) had increased signal amplitude changes within the posterior DMN network in schizophrenia patients compared to matched controls. These changes were detected within the RSN areas. Increased signal amplitude may reflect abnormally strong function within the network itself, without abnormal input from external sources.

The spatially shifted connectivity changes were either completely outside the network or in its border zones. Abnormality in network border zone connectivity was detected in the left inferior parietal lobule (BA 40) with left prominent ICA parcellation of CEN. Spatially, markedly aberrant connectivity of the right primary visual cortex (V1, BA 17R) was detected with the anterior CEN network – an area not usually belonging to the CEN at all. Spatially shifted connectivity can in theory mark abnormal functional input into the network’s activity and reflect external input into a functional unit of the brain. In addition, it may be that spatial shifting of the networks indicates abnormal spatial movement of networks recently detected using sliding window ICA.

### Mixed re-wirings between default mode and central executive networks

In our study, CEN represented an aberrant shifting of functional connectivity in the left inferior parietal lobule within IC20 and in the medial visual cortex, with IC1 that covered the anterior parts of CEN. Also, greater signal amplitude in the right dlPFC in IC11 was demonstrated. The dlPFC is usually regarded as a key hub of CEN (34, 67). Studies regarding dlPFC and CEN primarily report reduced connectivity between cortical and subcortical brain regions (23, 29, 30, 32, 34, 68). The abnormal shifting of the connectivity in ICA dual regression analyses might in part explain reductions seen in ROI-based studies; as functional activity moves to aberrant location, the activity within a spatially stable ROI may reduce. The increased signal amplitude then seems to oppose the reduced connectivity findings by others. However, according to preliminary analyses, it might be that the spatial stability of the network hubs might explain some key features detected in schizophrenia patients (69).

Tu et al. (34) used an ROI-based study to measure connectivity of key hubs of the CEN, and discovered reduced subcortical connectivity of several CEN key hubs, especially to the right caudate. Similarly, Su et al. (68) in their ROI-based study found reduced functional connectivity between the left dlPFC and bilateral caudate nucleus. Interestingly, in our study, the left caudate nucleus seems to be abnormally connected with the DMN rather than CEN. Strangely enough it becomes evident in the analysis focusing more on signal amplitude changes. Also Salvador et al. (18) reported an anterior node of DMN to have increased connectivity to the bilateral caudate, bilateral putamen, right pallidum, and posterior hippocampus, as well as to the right dlPFC. Taken together, it seems that functional connectivity between DMN, CEN, and subcortical regions, especially the caudate, seems to have abnormal wiring between the networks in schizophrenia.

Our result of aberrant connectivity to the right medial visual cortex suggests a dysfunction of visual sensory regions in schizophrenia; however, the alteration seems to be more closely related to the frontal parts of the CEN rather than anterior insula as suggested by Palaniyappan et al. (70). Reduced visual inflow could explain a wider brain connectivity locally. Following this, reduced connectivity of visual areas has been reported during task-performance (71, 72). A recent task-related fMRI study (73) showed that schizophrenia patients have reduced working memory with disturbed functional connectivity between prefontal and visual areas compared to healthy controls. Our results can be a resting-state connectivity manifestation of the same disease process, which causes the memory dysfunction.

### Hyperdopaminergic state and striatal rewiring

To this day, there is a sizeable amount of literature demonstrating dysfunction of dopaminergic neurotransmission in the striatum of schizophrenia patients, which also addresses connectivity changes in the caudate and key brain networks. However, direct proof is lacking for the role of dopamine in these functional impairments. Also, the exact location of dopamine dysfunction within the striatum remains to be addressed (74). Recent PET study findings suggested that schizophrenia is associated with elevated dopamine function in associative regions of the striatum, especially in pre-commissural dorsal caudate (75). The region found in the PET study is anatomically exactly the same as that found in our study being abnormally strongly connected to posterior DMN. Also, a recent semantic processing fMRI study has been shown to have diminished activation in the left caudate nucleus and greater activation in the left inferior frontal gyrus in a schizophrenic group compared to healthy controls (76). A decreased capacity to activate left caudate in semantic processing may be related to our finding of increased functional connectivity in a resting state. The study by Gradin et al. (36) showed reduced ventral striatal responses during reward and no-reward conditions, and, in addition, patients exhibited reduced functional connectivity between the midbrain and the right insula, correlating with increased severity of psychotic symptoms; this gives support for the argument that dopamine acts as a modulator. Hoptman et al. (77) in their resting-state study reported reduced *right* caudate resting-state signal amplitude of low-frequency fluctuations (ALFF) in schizophrenia patients. As stated earlier, we detected increased signal amplitude effects in *left* caudate nucleus with regards to aberrant connectivity to DMN. Taken together, our results and those of recent studies localize abnormal functional connectivity and brain activity in striatum, which is regarded as a dopaminergic system.

### Self-reflectiveness in schizophrenia

Recent meta-analyses on self-reflective processing occur in brain areas encompassing the dorsomedial and ventromedial prefrontal cortices, ACC, PCC, AI, inferior frontal gyrus, and temporo-parietal junction/angular gyrus/IPL (78, 79). Furthermore, introspective mental processes have been linked to a recruitment of the lateral prefrontal cortex (80), which is considered to be a portion of the CEN (26, 27). Jardri et al. (81) stated that the right IPL signal was found to correlate positively with the severity of first-rank symptoms in schizophrenia. After y-concat, IC43 (the angular variant of DMN) shows greater amplitude in the right inferior parietal lobule, and in bilateral medial frontal gyri (BA6), representing the involvement of DMN, and possibly implicating alterations in self-reflectiveness. Similarly to our findings, increased connectivity within the medial frontal gyrus in the DMN has been reported in several studies (22, 39, 82–84). Taken together, increased signal amplitude changes in DMN areas with increased signal connectivity may be related to altered introspective brain functions.

Increased connectivity in the medial frontal gyrus has also been reported in early-onset schizophrenia (85). Recently, also the familial risk to schizophrenia has been related to abnormal DMN connectivity in parietal regions (86). Interestingly, Guo et al. (87) recently also found a posterior parietal DMN abnormality in drug naïve first-episode subjects with schizophrenia in very similar areas. Our present results agree with these results that DMN is involved; however, the length of the disease (and treatment) seems to induce aberrancy of functional connectivity outside the DMN proper. Huang et al. (88) presented in their rsfMRI study of treatment-naïve first-episode schizophrenia patients that there are functional abnormalities of mPFC and putamen at an early stage of the disease. Neither Huang et al. nor Guo et al. detected abnormality in caudate, whereas we did; this may be an indication of a spreading disease involvement.

### Statistical significance and limitations

Aberrations in functional connectivity in subjects with schizophrenia were found throughout the brain after the initial dual regression, showing difference in 18 RSNs between groups. However, given the large number of RSNs tested, it is possible that some of these group differences could be false positive findings. The main focus of our discussion will, therefore, be restricted to the results obtained after the second level y-concat correction, adjusting for the risk of type 1 error (false positives) induced by increasing the number of independent components tested simultaneously.

Our findings at voxel level proved to be quite extensive and, more notably, the affected areas in our study seem to follow the lines of the results of previously conducted studies on schizophrenia (7, 9, 10, 12, 89). Our voxel-level results (without y-concatenation correction) indicate changes in the frontal parts and DMN of the brain, with both spatial and amplitude alterations in the prefrontal cortex, and amplitude changes in the inferior frontal gyrus and the frontal pole. Also, the involvement of the SN is more vividly displayed in our voxel-level findings. IC1, which portrays a part of the SN, shows a higher signal amplitude in the middle frontal gyrus. Our striatal findings consist of wider spatial activity and larger amplitude in the left nucleus caudate, also IC43 and IC49 demonstrate an amplitude difference in the cerebellum.

The fact that our results diminished to four ICs parcellations of two main RSNs displays the relatively stringent thresholding effect of the second level y-concat correction. Y-concatenation, like many other methods controlling for false positives, emphasizes the spatial extent of clustered group differences over sparse activity. In addition to the task of removing the risk of type 1 error (false positives) induced by the use of a high number of components tested simultaneously, a number of “true” results could also be affected. For example, there are differences between groups in IC 46 (left DLPFC) that are spatially sparse and fail to survive y-concat, while its mirror component IC 11 (right DLPFC) had a spatially tight cluster, with 20 voxels surviving y-concatenation.

In this study, we are not measuring correlation coefficients between anatomical regions, and therefore, we are not talking about connectivity between anatomical regions so much. ICA depicts RSN networks as a whole, and not based on functional connectivity strength between areas. ICA rather looks at the statistical independence of functional parcellations compared to other signal sources in the BOLD data as a whole. DMN, like any network representation after dual regrression, can be analyzed for areas, which show abnormal connectivity to that given network. ICA can also show if connectivity strength is abnormal in a network. One could also talk about “belongliness” of an area with a network in principle. Therefore, we tend to prefer to talk about areas with aberrant connectivity to a network rather than between some specific regions *per se*.

The results of spatial shifting and amplitude changes are not explicit in nature. The default mode and CEN, especially those showing the most prominent changes, present differences in both signal amplitude analysis and anatomical shifting to aberrant connectivity areas commonly considered to be outside the network itself. For example, caudate nuclei showed increased signal intensity based connectivity within the DMN, even though most changes in DMN were related to signal amplitude changes within the network proper. It also seems that the variance normalization effects in dual regression tend to *emphasize* the differences toward being either spatial or signal amplitude in nature. This was also stated in original work by Allen and co-workers (50). Therefore, the results may in some cases be somewhat overlapping, it might be beneficial to use caution in order not to over-interpret the ICA dual regression based group differences strictly to be either spatial or signal amplitude in origin. Also, novel ultra-fast fMRI sequences may provide a more comprehensive picture of the events in the future.

Antipsychotic drugs are a difficult and common covariate in this field of study, and they have exhibited changes in DMN (90); the results of a motor task fMRI study suggested that antipsychotics reduce activation in motor (cortical and subcortical including caudate) and DMNs in schizophrenia patients (91). Antipsychotic treatment could be a factor confounding the findings, especially in nucleus caudate, but also brain-wide. However, no effect of medication was found in our preliminary studies when using the medication as a covariate with the same study sample (results not shown here). Deeper analysis on duration, compliance, and dose needs to be performed in further studies in this regard.

## Conclusion

The most persistent differences between schizophrenia patients and healthy controls in the human connectome were detected in default and central executive networks. These networks portrayed mixed, both anatomically shifted and altered signal strength alterations in functional connectivity between groups. Notably, schizophrenia patient data always portrayed increases in functional connectivity, never the controls. Our results suggest that abnormalities in schizophrenia consist of mixed rewiring alterations focusing in left caudate, parietal lobule, and dlPFC in default mode and central excecutive networks.

## Conflict of Interest Statement

The authors declare that the research was conducted in the absence of any commercial or financial relationships that could be construed as a potential conflict of interest.

## Supplementary Material

The Supplementary Material for this article can be found online at http://www.frontiersin.org/Journal/10.3389/fpsyt.2015.00026/abstract

## Abbreviations

ALFF, amplitude of low-frequency fluctuations; BOLD, blood oxygen level dependent; CEN, central executive network; dACC, dorsal anterior cingulate cortex; dlPFC, dorsolateral prefrontal cortex; DMN, default mode network; fMRI, functional magnetic imaging; FPN, frontoparietal network; FSL, FMRIB Software Library; IC, independent component; ICA, independent component analysis; IPL, inferior parietal lobule; mPFC, medial prefrontal cortex; PCC, posterior cingulate cortex; PET, positron emission tomography; rAI, right anterior insula; rsfMRI, resting-state fMRI; RSN, resting-state network; SN, salience network; TPN, task-positive frontoparietal network; y-concat, y-concatenation.
