# Supplementary material for: Aberrant Functional Connectivity in the Default Mode and Central Executive Networks in Subjects with Schizophrenia – A Whole-Brain Resting-State ICA Study
Source: Front Psychiatry. 2015 Feb 26;6:26. doi: 10.3389/fpsyt.2015.00026 (PMC4341512; doi:10.3389/fpsyt.2015.00026)
Supplement: Supplementary file 1 [file Presentation_1.PDF]

Supplementary data

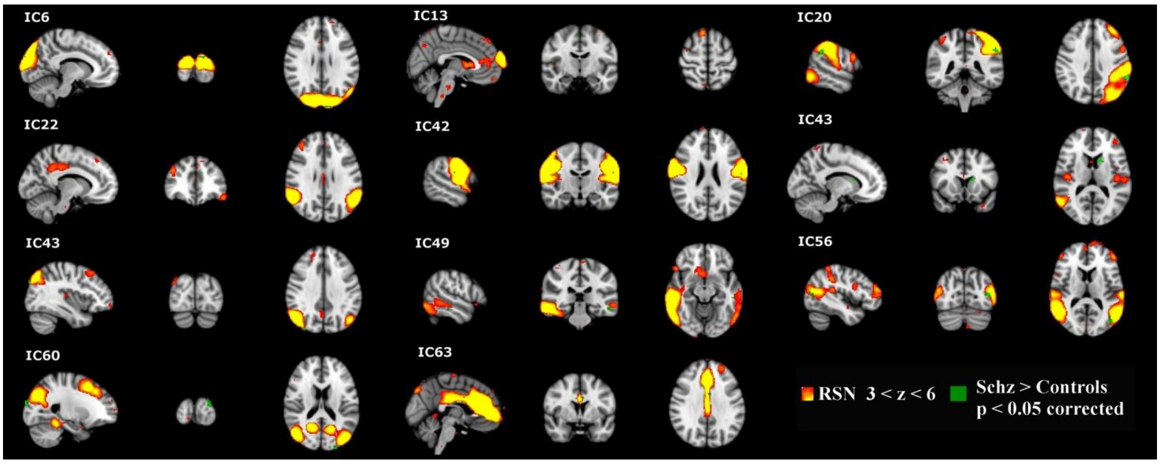

Supplementary Figure 1. Initial dual regression used with non-normalized variance (des_norm=0) showing anatomically shifted differences in the functional connectivity in RSN's.

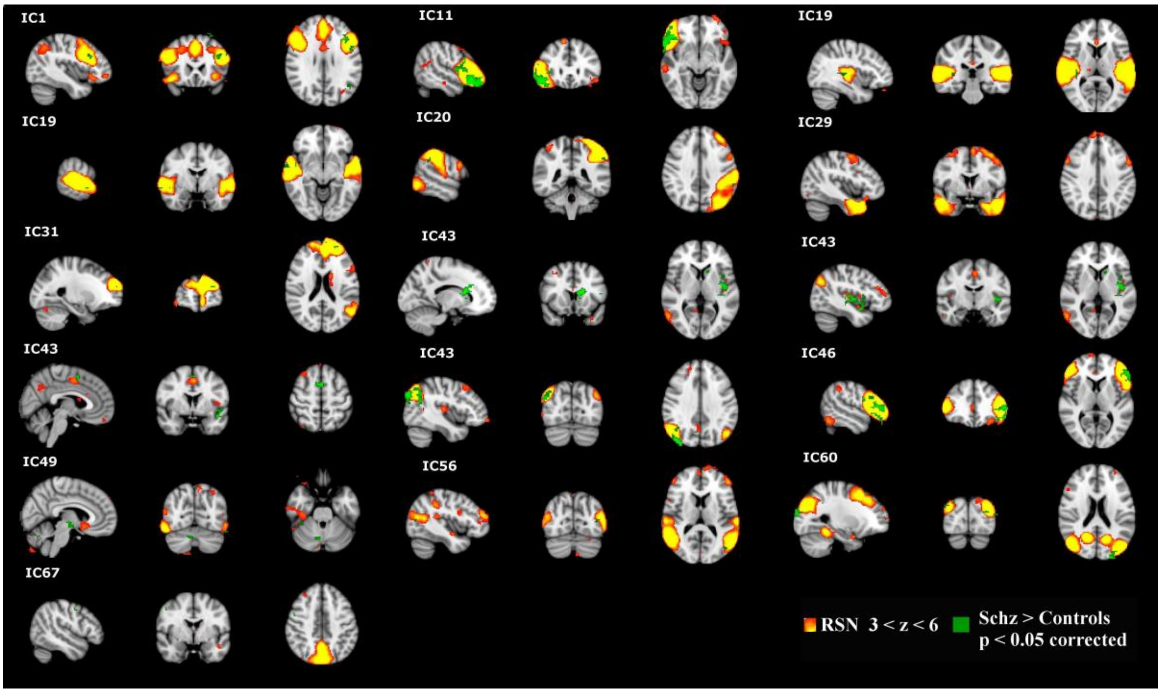

Supplementary Figure 2. Initial dual regression used with normalized variance (des_norm=1) showing BOLD signal amplitude changes in the RSN's.

			Anatomical location					Max coordin		
Spatial	IC #	vox		mean T-score	Std	min	max	X	Y	Z
	6	6	Visual cortex, BA 18 L	3.63	0.19	3.50	4.01	26	7	25
	13	2	Juxtapositional lobule cortex	4.33	0.09	4.28	4.40	21	29	33
	20	16	Left inferior parietal lobule	3.91	0.58	3.31	5.31	38	20	26
	22	1	Left paracingulate gyrus	4.66	-	4.66	4.66	25	41	25
	42	14	Left postcentral gyrus	3.30	0.29	2.92	3.94	39	32	20
	43	18	Left caudate	3.93	0.42	3.29	4.68	27	36	22
	49	4	Left middle temporal gyrus	3.89	0.16	3.75	4.11	35	24	14
	56	17	Left lateral occipital cortex, V5 L	3.33	0.32	2.98	4.04	35	12	22
	60	26	Visual cortex, BA 17 L	3.55	0.43	3.03	4.52	38	36	16
	63	2	Anterior cingulate gyrus	4.30	0.20	4.16	4.44	24	32	26
Amplitude										
	1	137	Left middle frontal gyrus	3.13	0.45	2.41	5.68	18	14	21
	11	300	Right inferior frontal gyrus	2.63	0.40	2.15	4.48	9	38	13
	19	31	Right auditory cortex	3.08	0.32	2.68	3.86	37	28	13
	20	8	Left inferior parietal lobule	3.94	0.49	3.56	4.90	20	44	27
	29	7	Left precentral gyrus	4.16	0.56	3.52	5.09	16	2	14

									3	
	31	27	Left frontal pole	3.93	0.55	3.04	5.32	24	38	33
	43	309	Left caudate	2.86	0.56	2.14	4.86	14	10	26
	46	188	Left Broca’s area BA45	2.71	0.37	2.27	4.16	37	39	16
	49	67	Cerebellum	2.99	0.35	2.37	3.90	20	14	11
	56	6	Left lateral occipital cortex	3.39	0.26	3.16	3.75	33	13	21
	60	45	Left lateral occipital cortex	3.40	0.45	2.66	4.85	38	35	16
	67	3	Right premotor cortex	4.23	0.12	4.09	4.32	10	31	29

Supplementary Table 1. Group comparison of independent components after initial dual regression. Co-ordinates indicating the centroid of the cluster of altered brain activity.
